# Probiotics for constipation in Parkinson’s: A systematic review and meta-analysis of randomized controlled trials

**DOI:** 10.3389/fcimb.2022.1038928

**Published:** 2022-11-10

**Authors:** Shao Yin, Fengya Zhu

**Affiliations:** ^1^ Hospital of Chengdu University of Traditional Chinese Medicine, Chengdu, China; ^2^ Traditional Chinese Medicine Department, Zigong First People’s Hospital, Zigong, China

**Keywords:** Parkinson, probiotics, constipation, systematic review, meta-analysis

## Abstract

**Background:**

Parkinson’s disease (PD)-related constipation may affects both disease occurrence and disease progression. Probiotics, as a potential therapeutic intervention, have attracted the attention of researchers, but the evidence of their efficacy and safety has not been systematically reviewed.

**Aim:**

A systematic review and meta-analysis of randomized controlled trials of probiotics in the treatment of PD constipation was conducted to determine the efficacy and safety of probiotics in the treatment of PD constipation.

**Methods:**

Four databases (The Cochrane Central Register of Controlled Trials, Embase, PubMed, and Web of Science) were searched from their establishment to June 1, 2022. We included randomized controlled trials of probiotics for the treatment of constipation in patients with PD, with probiotics in the experimental group and a placebo, another treatment, or no treatment in the control group. The primary outcome was the number of bowel movements per week. Secondary outcomes included nonmotor symptoms (NMS), gut transit time (GTT), abdominal pain, abdominal distention, constipation, and quality of life scores. Stata15.1 was used to generate a summary of the data and perform a descriptive analysis if necessary. The GRADE tool was used to assess the quality of the evidence and the Cochrane guidelines to assess the risk of bias for each study.

**Results:**

Finally, four qualified RCTs were included, comprising 287 participants. Compared with the control group, probiotics could effectively increase the frequency of defecation per week in PD patients (WMD = 1.02. 95%CI: 0.56–1.48, and *P* < 0.00001), but the heterogeneity was high, and the quality of the evidence was low. There was no significant difference in average stool consistency between patients with PD treated with probiotics and those given a placebo in (WMD = –0.08. 95%CI: –1.42–1.26, and *P* = 0.908). In addition, the results suggested that probiotics have no obvious effect on additional indicators of gastrointestinal dysfunction, such as GTT, abdominal pain, and abdominal distension, and there is insufficient evidence on their ability to improve NMS and Parkinson’s disease Questionnaire 39 summary indices (PDQ39-SI). Safety issues should be carefully explained.

**Conclusion:**

There is insufficient evidence supporting the use of probiotics to treat constipation in patients with PD. Taking all the results together, probiotics have potential value in the treatment of PD-related constipation.

**Systematic Review Registration:**

PROSPERO CRD42022331325.

## Introduction

Parkinson’s disease (PD) is the second most common neurodegenerative disorder after Alzheimer’s disease, and dyskinesia is a major feature of PD. In fact, a range of nonmotor symptoms (NMS) associated with autonomic nervous dysfunction, especially dysfunction of the gastrointestinal tract (Borek et al., 2006; Cloud and Greene, 2011; Fasano et al., 2015), may occur at all stages of PD (Chaudhuri and Schapira, 2009) and may even be closely related to the pathogenesis of PD (Braak et al., 2003; Holmqvist et al., 2014). NMS has a significant negative impact on clinical care and health-related quality of life (Hr-QoL) in patients with PD (Li et al., 2010; Lyons and Pahwa, 2011).

Constipation is one of the most common NMS in PD patients with autonomic system and gastrointestinal disorders (Sakakibara et al., 2003; Verbaan et al., 2007; Gao et al., 2011), even before the onset of motor symptoms (Abbott et al., 2001; Savica et al., 2009). It affects about 50%–80% of PD patients (Ashraf et al., 1997; Verbaan et al., 2007). The evidence shows that constipation is related to the duration and severity of PD (Krogh et al., 2008), and the frequency and severity of constipation are accelerated by the progression of PD (Edwards et al., 1993). Clearly, constipation and PD have reciprocal effects (Fu et al., 2022).

PD-related constipation is an active research field. Various studies have evaluated different drugs for the treatment of PD-related constipation, but there is no clear guideline recommendation so far (Poirier et al., 2016). Clearly, it is still necessary to explore effective and safe emerging drugs (Pohl et al., 2008). Previous studies have shown that probiotics can significantly improve the stool consistency and bowel habits of PD patients (Cassani et al., 2011); increase the number of complete bowel movements per week (CBM) and gut transit time (GTT) (Barichella et al., 2016; Ibrahim et al., 2020; Tan et al., 2021); and reduce abdominal pain, abdominal distension, and incomplete emptying in PD patients (Perez-Lloret et al., 2013; Georgescu et al., 2016).

However, evidence for the positive effects of probiotics on PD constipation is inconclusive. Therefore, this systematic review and meta-analysis included data from the results of several clinical trials evaluating the efficacy and safety of probiotics in the treatment of PD-related constipation. We aim to provide a comprehensive update of the clinical data for evidence-based guideline development.

## Methods

### Eligibility criteria

This study included all randomized controlled trials of probiotics in the treatment of PD-related constipation. PD participants meet internationally recognized diagnostic criteria and had constipation or gastrointestinal dysfunction. The experimental group was treated with probiotics, while the control group was treated with a placebo, other treatments, or no intervention. The main outcome was the number of bowel movements per week; secondary outcomes included average stool consistency, NMS, GTT, abdominal pain, abdominal distension, constipation, and quality of life scores.

Reviews, conference papers, comments, animal studies, retrospective studies, case-control studies, and self-controlled studies were excluded. RCTs that did not include constipation-related outcomes were also excluded.

### Search strategy

RCTs were searched in The Cochrane Central Register of Controlled Trials, Embase, PubMed, and Web of Science from inception to June 1, 2022. In addition, a list of references included in the study was manually searched to identify relevant trials. There were no restrictions on language, year of publication, etc. Grey literature and data on the research registry platform were not within the scope of the search because we do not have access to these. Detailed search strategies are available in the **Supplementary Materials**.

### Study selection

According to the strict retrieval strategy, reviewers used Endnote X9 and manual procedures to delete duplicate documents. Initial study selection was performed according to the title and abstract, followed by full-text reading to determine the final included studies. Two reviewers completed the literature search and screening independently. Any disagreement between the two reviewers was resolved by discussion. If no agreement was reached, the final decision was made by a third reviewer.

### Data extraction

Data extraction was performed independently and cross-checked by two examiners according to a standardized form developed in advance. The main contents included the publication year, first author, country, study design, participants (age, sex), sample size, intervention details (formulation, dose, duration), results, etc. Any disagreement between the two reviewers was resolved by discussion. If no agreement was reached, the final decision was made by a third reviewer.

### Data synthesis and statistical analysis

A meta-analysis was conducted on the same outcome indicators in two or more RCTs. Continuous variables were represented by the weighted mean difference (WMD) and 95% confidence interval (CI), a result was considered statistically significant at P < 0.05, and if I^2^ ≥ 50%, a random effect model was used, and sensitivity analysis was conducted to observe the stability of the results. Due to the small number of RCTs included in this study, we did not test for publication bias. For individual outcome measures, data were summarized, and descriptive analysis was performed.

### Assessment of the risk of bias and quality of evidence

A risk of bias assessment was conducted for each RCT according to the Cochrane Handbook. The evaluation areas included random sequence generation, allocation concealment, blinding of participants and personnel, blinding of outcome assessment, incomplete outcome data, selective reporting, and other sources of bias. Each area was rated as a high, low, or unclear risk. Evaluation of the quality of evidence against Grading of Recommendations Assessment, Development, and Evaluations (GRADE) consists of five main factors: risk of bias, inconsistency, imprecision, indirectness, and publication bias. All evaluations were conducted independently by two reviewers, with unresolved differences determined by a third reviewer.

## Results

### Results of literature search and selection

**Figure 1:**
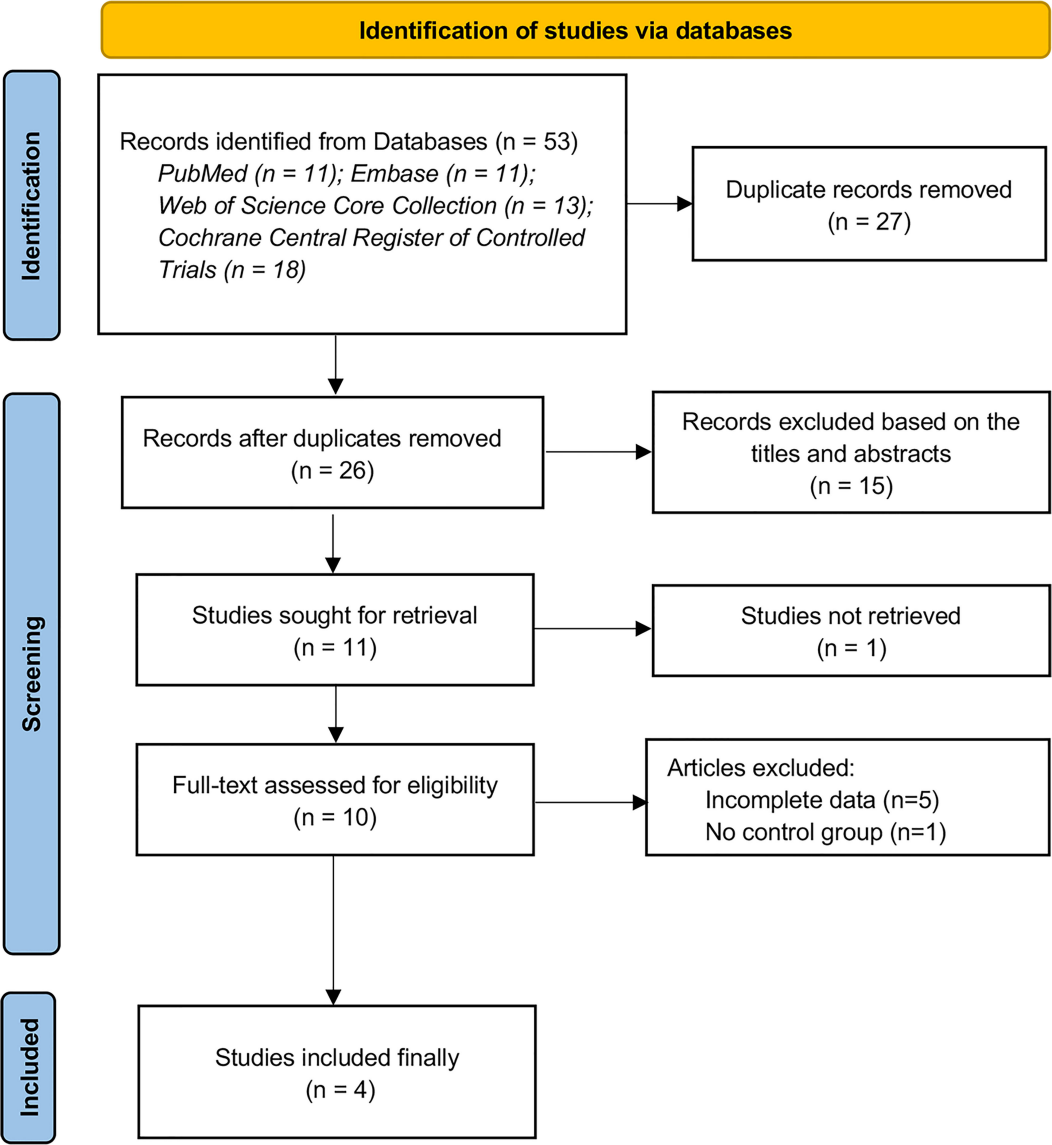
Flow chart of study selection.

We retrieved 53 related articles from 4 databases and removed 27 duplicates. Then, four qualified studies were included through title, abstract, and full text evaluation. The detailed flow chart is shown in **Figure 1**. The excluded list in the “full-text assessed for eligibility” phase are outlined in the **Supplementary Materials**.

### Characteristics of included studies

Four RCTs with 287 participants were included in the study, which was conducted in Italy, Romania, and Malaysia between 2016 and 2021. Participants were over 60 years old on average, there were more men than women, and the duration of treatment ranged from four weeks to three months. Except for one study in which trimebutine was used in the control group, all the participants in control groups took a placebo with the same characteristics as the treatment given to the intervention group but without probiotics. Three RCTs showed adverse reactions, mainly manifested as abdominal pain, abdominal distension, and dizziness, and the experimental group was larger than the control group. Detailed literature features are shown in **Table 1**.

**Table 1:** Detailed literature features.

Included studies	Country	Sample size(I/C)	Age [y, mean (SD)] (I/C)	Sex (male/female) (I, C)	Intervention	Comparison	Duration	Adverse events (I/C)
Barichella M, 2016	Italy	80/40	71.8(7.7)/69.5(10.3)	41/39, 24/16	Fermented milk containing probiotics and prebiotic fiber (125 g) Including the following strains: Streptococcus salivarius subsp thermophilus, Enterococcus faecium, Lactobacillus rhamnosus GG, Lactobacillus acidophilus, Lactobacillus plantarum, Lactobacillus paracasei, Lactobacillus delbrueckii subsp bulgaricus, and Bifidobacterium (breve and animalis subsp lactis)/Qd	Placebo (a pasteurized, fermented, fiber-free milk)/Qd	4 w	1(abdominal pain and bloating))/1(abdominal pain and bloating)
Georgescu D, 2016	Romania	20/20	69.80 (5.64)/75.65(9.66)	10/10, 7/13	Mixture of two lactic bacteria: Lactobacillus acidophilus and Bifidobacterium infantis, 60 mg/Bid	Trimebutine, 200mg/Tid	3 m	None
Ibrahim A, 2020	Malaysia	27/28	69.0/70.5	16/9, 17/10	Probiotic (Hexbio®) in orange flavouring containing microbial cell preparation of (MCP®BCMC®) at 30 x 109 colony forming units (CFU), 2% fructo-oliogosaccharide (FOS), and lactose. The microbial composition of the probiotics were: Lactobacillus acidophilus (BCMC® 12130)– 107mg, Lactobacillus casei (BCMC® 12313) -107mg, Lactobacillus lactis (BCMC® 12451)-107 mg, (BCMC® 02290) -107mg, Bifidobacterium infantis (BCMC® 02129) -107mg and Bifidobacterium longum (BCMC® 02120)-107mg./Bid	Granulated milk of similar appearance to the probiotics containing lactose without fructo-oligosaccahride or microbial cells in orange flavouring/Bid	8 w	4(abdominal bloating, n=2; dizziness, n=2)/0
Tan AH, 2021	Malaysia	34/38	63.1/61.5	42/29, 26/11	Probiotic capsule, contained 10 billion colony forming units (CFU) of eight different commercially available bacterial strains (Lactobacillus acidophilus, Lactobacillus reuteri, Lactobacillus gasseri, Lactobacillus rhamnosus, Bifidobacterium bifidum, Bifidobacterium longum, Enterococcus faecalis, Enterococcus faecium)/Qd	Placebo capsul: containing an inactive substance (maltodextrin)/Qd	4 w	1(lethargy)/0

I, intervention group; C, comparison group; F, Frequencies; m, months; y; w, week; Qd, Once a day; Bid, Twice a day; Tid, Three times a day.

At the same time, we summarized the baseline information of severity of PD symptoms of participations and PD drugs used in the three RCTs included in the meta-analysis. Ibrahim et al. (2020) included idiopathic PD patients in Hoehn and Yahr stages 1–4, The proportion of participations with stage 3 and below in the experimental group and the control group was 59.3%/64.3%, and the proportion of participations with levodopa in the two groups was 92.6%/89.3% and dopamin agonist were 63%/57.1%; Barichella et al. (2016) also included idiopathic PD patients in Hoehn and Yahr stages 1–4, the proportion of participations with stage 3 and below in the experimental group and the control group was 76.3%/75%, and the proportion of participations who received dopamine-agonist therapy in the two groups was 63.8%/62.5%, and the daily dose of levodopa in the two groups was 691mg ± 315mg/624mg ± 289mg. Tan et al. (2021) used Movement Disorder Society Unified Parkinson’s Disease Rating Scale to evaluate the severity of participations, the score of experimental group and control group was 27.9 ± 12.8/27.5 ± 12.6. The comparison of the proportion of participations taking drugs in the two groups was as follows: levodopa (97.1%/97.4%), caudate agonist (38.2%/39.5%), and anticholinergics (17.6%/13.2%). There was no significant difference in the severity of PD symptoms of participations and PD drugs used between the experimental group and the control group in the three RCTs.

### Risk of bias

**Figure 2:**
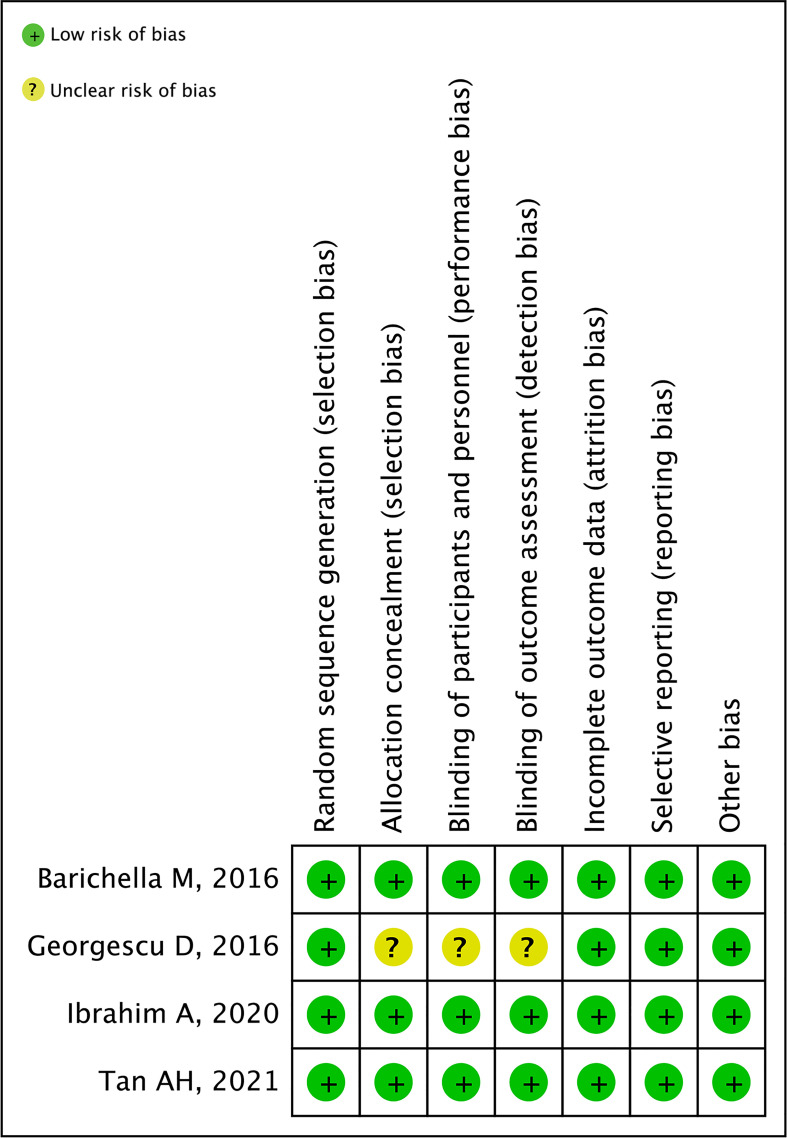
The risk of bias of studies.

We assessed the risk of bias for four RCTs using the Cochrane Collaboration Handbook. The study by Georgescu et al. (2016) did not mention the allocation of hidden schemes, and the blinding was not sufficiently informative, so its risk of bias was rated as unclear. The risk of bias of all other studies was rated as low (**Figure 2**).

### Results of the meta-analysis

**Figure 3:**
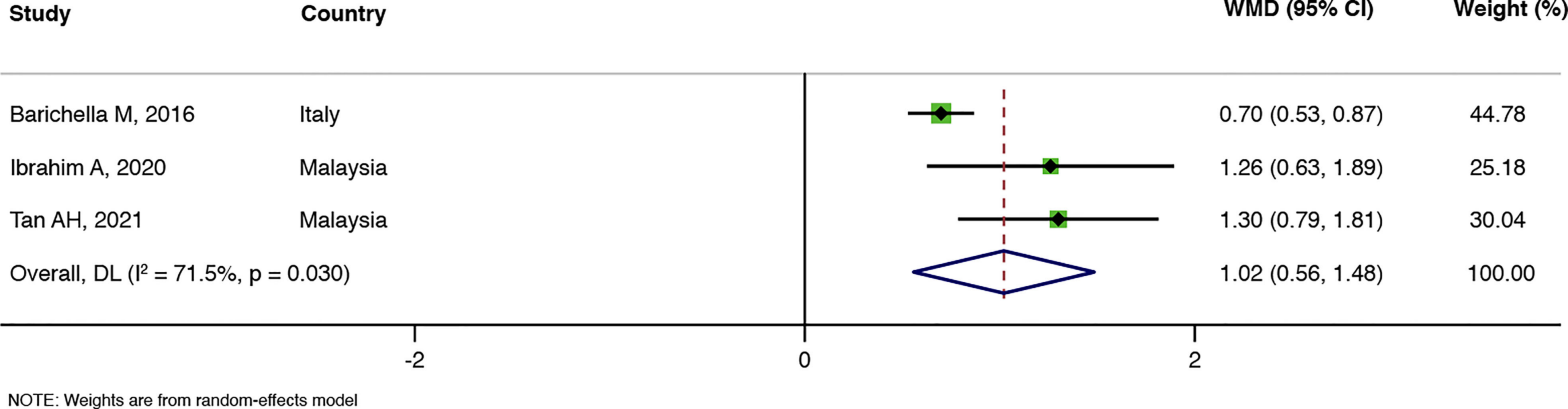
Meta-analysis of the number of bowel movements per week.

**Figure 4:**
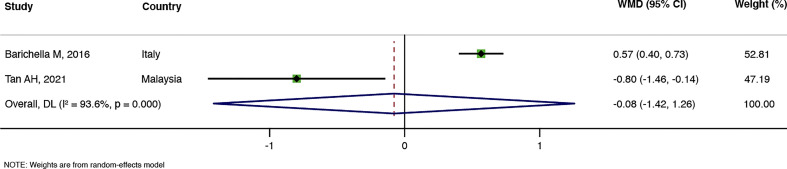
Meta-analysis of average stool consistency.

The number of bowel movements per week was reported in three RCTs (Barichella et al., 2016; Ibrahim et al., 2020; Tan et al., 2021). A Meta-analysis suggested that probiotics could effectively increase the number of bowel movements per week in PD patients compared with the control group (WMD = 1.02, 95%CI: 0.56–1.48, and *P* < 0.00001), but the heterogeneity was high (I^2^ = 71.5%, P = 0.030), as shown in **Figure 3**. Two RCTs (Barichella et al., 2016; Tan et al., 2021) calculated the changes in the average stool consistency in PD patients, but the results were inconsistent, and meta-analysis results showed no statistically significant difference between the probiotic group and the control group (WMD = –0.08, 95%CI: –1.42 to 1.26, and *P* = 0.908), with high heterogeneity (I^2^ = 93.6%, P = 0.000). Detailed results are shown in **Figure 4**.

### Summary of the outcomes

Georgescu et al. (2016) assessed gastrointestinal function (GI) in the NMS of PD patients. The results showed that probiotics showed the potential to relieve abdominal pain and abdominal distention in PD patients but had no significant effect on relieving constipation symptoms in PD patients. Overall, there was no statistically significant difference between the probiotic group and the trimebutine group. The results of a study by Ibrahim et al. (2020) showed that probiotics can shorten GTT and reduce the Non-motor Symptom Scale (NMSS) score in PD patients. There was a potential improvement in the scores on Parkinson’s disease Questionnaire 39 Summary Indices (PDQ39-SI), but there was no significant difference compared with the placebo group. Tan et al. (2021) showed that probiotics could significantly relieve the degree of constipation in PD patients. More detailed results are shown in **Table 2**.

**Table 2:** Summary of the outcomes.

Study	Sample size(I/C)	Outcomes	Intervention			Comparison		
			Baseline[mean (SD)]	After treatment [mean (SD)]	*P*-value	Baseline[mean (SD)]	After treatment [mean (SD)]	*P*-value
Georgescu D, 2016	20/20	Abdominal pain*	1.45 (0.51)	1.05 (0.69)	0.00432	1.55 (0.51)	0.6 (0.52)	<0.0001
		Bloating*	1.4 (0.5)	0.3 (0.47)	<0.0001	1.6 (0.5)	0.45 (0.51)	<0.0001
		Constipation*	1.35 (0.49)	1.15 (0.49)	0.2040	1.5 (0.51)	0.85 (0.67)	0.0014
Ibrahim A, 2020	27/28	GTT	125.26 (54.81)	77.32 (55.35)	<0.001	128.46 (53.68)	113.54 (61.54)	0.093
		NMSS	63.66 (35.22)	47.5 (30.07)	<0.001	71.6 (42.34)	63.5 (44.92)	0.007
		PDQ39-SI	33.1 (25.59)	26.87 (26.14)	0.013	40.1 (28.12)	36.17 (21.01)	0.341
Tan AH, 2021	34/38	Constipation severity score (0-15) †	8.4 (2.3)	5.2 (3.39)	–	7.5 (2.7)	5.9 (2.89)	–

GTT, Gut transit time; NMSS, Non motor Symptom Scale; PDQ39-SI, Parkinson’s disease Questionnaire 39 summary indices.

^*^A scoring of the symptoms was set using a scale from 0 to 3, with 0 indicating no symptoms, 1 indicating mild symptoms, 2 indicating moderate symptoms, and 3 indicating severe symptoms.

^†^Based on the constipation severity questionnaire adapted from Rome IV criteria (higher scores indicate worse severity)

### Adverse reactions

A total of 7 adverse reactions were noted in the three RCTs, 6 of which occurred in the probiotic group, mainly abdominal pain, abdominal bloating, dizziness, and lethargy. Among them, Ibrahim et al. noted that side effects such as abdominal bloating and dizziness were transient, and symptoms resolved when probiotics were discontinued. No serious adverse reactions related to probiotic treatment were observed.

### Sensitivity analysis and publication bias

We performed sensitivity analysis on the meta-analysis results of the number of bowel movements per week, and the results were stable **(** **Supplementary Materials** **)**. Due to the small number of RCTs included in this study, we did not conduct a publication bias assessment.

### Grade

Due to the high heterogeneity and small sample size in the meta-analysis of the number of bowel movements per week and average stool consistency in Parkinson’s patients, the evidence level of the results was rated as low, as shown in **Table 3**.

**Table 3:** The result of the GRADE.

Outcomes	No of Participants(studies) Follow up	Quality of the evidence (GRADE)	Relative effect(95% CI)	Anticipated absolute effects	

				Risk with Control	Risk difference (95% CI)
**The number of bowel movements per week**	240 (3 studies)	⊕⊕⊝⊝ **LOW** ^1,2^ due to inconsistency, imprecision			The mean the number of bowel movements per week in the intervention groups was 1.02 (0.56 to 1.48)
**Average stool consistency**	192 (2 studies)	⊕⊕⊝⊝ **LOW** ^1,2^ due to inconsistency, imprecision			The mean average stool consistency in the intervention groups was -0.08 (-1.42 to 1.26)

CI: Confidence interval.

GRADE Working Group grades of evidence

High quality: Further research is very unlikely to change our confidence in the estimate of effect.

Moderate quality: Further research is likely to have an important impact on our confidence in the estimate of effect and may change the estimate.

Low quality: Further research is very likely to have an important impact on our confidence in the estimate of effect and is likely to change the estimate.

Very low quality: We are very uncertain about the estimate.

^1^ Serious inconsistency due to moderate heterogeneity with 50% < I2 and P value (chi-square test) < 0.10.

^2^ Very serious imprecision due to the small sample size (< 400 individuals).

## Discussion

A total of four RCTs evaluating probiotics for PD constipation were included in this systematic review. A meta-analysis showed that probiotics increased the number of bowel movements per week in PD patients but had no effect on average stool consistency. Although the sensitivity analysis showed that the results were stable, subgroup analysis could not be carried out due to the small number of RCTs included, and the source of heterogeneity could not be found. Different diagnostic criteria, different doses and types of probiotics taken, and the small number of included literatures may be the reasons for the high heterogeneity. High heterogeneity in the results and the small sample size of the meta-analysis resulted in a low quality of evidence. In addition, receiving probiotics or a placebo showed no significant difference in terms of alleviating abdominal pain, abdominal distention, GTT, and other gastrointestinal disorders in PD patients.

The gut-brain axis refers to the dynamic bidirectional interaction between the intestinal flora and the central nervous system. The interaction between the central nervous system and the gut mainly connects peripheral intestinal function to the emotional and cognitive brain centers through various neuro-immune-endocrine mediators (Naomi et al., 2021). An imbalance in the intestinal flora affects the occurrence and progression of neurodegenerative diseases and mental disorders, while supplementation with dietary fiber and probiotics can improve various cognitive functions (Barbosa and Vieira-Coelho, 2020; Barrio et al., 2022). Despite the popularity of probiotics as a treatment for neurodegenerative diseases in recent years, the results of studies on probiotics have been inconsistent.

The FAO/WHO defines probiotics as “living microorganisms beneficial to the health of the host when ingested in an appropriate amount” (Hill et al., 2014). A study summarized the evidence of the relationship between the intestinal microflora, cognitive function, and dementia pathology in the elderly, and its conclusion supported the impact of intestinal microorganisms on cognitive function. In animal studies, prebiotics and probiotics had a positive effect on cognitive function (Neta et al., 2022; de Rijke et al., 2022), but the existing evidence is insufficient to support a clinical application (Ticinesi et al., 2018).

Gastrointestinal tract is closely related to the central nervous system, environmental pathogens may enter the central nervous system through the vagal connections in the gut, and eventually accelerate the progression of PD (Travagli et al., 2020). Constipation is a prevalen non-motor symptom in PD, its underlying mechanism and pathophysiology is complex, such as accumulation of alpha-synuclein originate from the myenteric plexus in the intestine may be one of the reasons (Fasano et al., 2015; Barrenschee et al., 2017). At the same time, the use of anti-parkinsonism drugs can also result in slow colonic transport or puborectalis dyssynergia and aggravate constipation symptoms (Stocchi and Torti, 2017). Moreover, PD patients are associated with lower short chain fatty acids (SCFAs), which have anti-inflammatory properties and are essential for gut mucosal lining repair, regulation of intestinal nervous system activity, and enhancement of gut motility (Unger et al., 2016; Aho et al., 2021). The mechanism by which probiotics improve PD constipation may be through the increase of SCFAs and mucin production in the gut thereby repairing the gut mucosal lining and enhancing gut motility (Dimidi et al., 2017; Suez et al., 2019). Whether and to what extent probiotics, while relieving constipation, also slow the progression of PD, remains to be investigated.

The authors of several systematic reviews and meta-analyses evaluating the use of probiotics for Alzheimer’s disease (AD), mild cognitive impairment (MCI), and PD believe that probiotics and synbiotics supplements improve cognitive function in patients with AD, while no positive effect was seen in other biomarkers of oxidative stress or lipid profiles. Only insulin resistance could be improved in patients with AD (Krüger et al., 2021; Li et al., 2021), and dietary probiotics could improve cognitive function in MCI patients, but in another study, the effect on AD patients was limited (Zhu et al., 2021). However, studies by Leta et al. (2021) highlighted that probiotic therapy can increase glucose metabolism, reduce peripheral and central inflammatory responses (e.g., reduction of interleukin-6 (IL-6), hs-CRP, and tumor necrosis factor -α (TNF-α) in PD patients, and increase motor and non-motor function. The results of a meta-analysis by Xiang et al. (2022) suggest that probiotics can enhance the cognitive function of AD and MCI patients and improve the gastrointestinal symptoms of PD patients, for example, by relieving abdominal pain, abdominal distention, and constipation and increasing the number of bowel movements per week, with no significant effect on stool consistency. In addition, probiotics can also reduce biomarkers of inflammation and oxidative stress. The results of gastrointestinal symptoms were similar to those of this study.

Based on the gut-brain axis connection, patients with neurological diseases have a much higher risk of intestinal dysfunction. How to effectively manage intestinal disorders has always been a focus of the medical field, while intestinal management in the past was empirical with very little research basis (Coggrave et al., 2006). In the updated Cochrane Systematic Review, interventions to address constipation remain limited, and the quality of the evidence is very low due to differences in intervention and control approaches. At present, common methods to improve constipation mainly include catharsis, abdominal massage, electrical stimulation, an anticholinesterase anticholinergic drug combination (neostigmine glycopyrrolate), anal flushing, oral carbonated water, and lifestyle modification (Coggrave et al., 2014). Probiotic therapy has been well documented in patients with simple functional constipation, with multistrain probiotics significantly reducing GTT, increasing stool removal frequency, and improving stool consistency. Therefore, probiotics are considered safe and natural remedies for the relief of functional constipation in adults (Zhang et al., 2020).

However, The International Parkinson and Movement Disorder Society (MDS) Evidence‐Based Medicine (EBM) Committee only recommended Macrogol, Lubiprostone, and Probiotics/Prebiotic fibers as three medicines/foods used to treat PD-related constipation (Hatano et al., 2022). Chronic constipation is the earliest symptom of PD prodrome and one of the universal NMS in PD (Kalia and Lang, 2015). This systematic review focused on the evaluation of probiotics for the treatment of constipation in PD patients. Probiotics increased the number of weekly defecations in PD patients compared with a placebo, but with high heterogeneity and a low quality of evidence. Our results also suggest that probiotics have no significant beneficial effect on stool consistency, GTT, NMSS, and PDQ39-SI, and there is no clear evidence that probiotics have a significant effect on additional symptoms of gastrointestinal dysfunction, such as abdominal pain and bloating. In terms of safety, clinical studies have reported adverse reactions such as abdominal pain and abdominal distention in the probiotic group. In fact, gastrointestinal dysfunction in PD patients, as one of the common NMS, may include clinical symptoms such as abdominal pain and abdominal distension. Whether adverse reactions are caused by drugs requires careful consideration. In addition, the included studies also reported two adverse reactions of lethargy and dizziness in the probiotics group. Although the authors indicated that the symptoms disappeared after the cessation of probiotics and no serious adverse reactions occurred, the safety of probiotics still needs to be verified in subsequent studies. Meanwhile, clinical studies are limited, the overall sample size is small, and whether probiotics synthesized by different strains have different effects on intestinal function still needs further research. A large sample size and high-quality clinical evidence are still the top priority to clarify the efficacy and safety of probiotics in the treatment of PD-related constipation.

### Strengths and limitations

The problem of constipation in PD patients is closely related to the progression of their own disease. With the gradual emergence of probiotics, it is clearly important to determine the effectiveness and safety of probiotics on PD-related constipation for clinical selection. Here, we must point out that the systematic review has some limitations. First, the number of clinical studies was limited. We only included four RCTs involving 287 participants and only conducted a meta-analysis on the two main results, the number of bowel movements per week and thin stool consistency, which had high heterogeneity. Secondly, due to the small number of RCTs included in this study, the composition, dosage, and frequency of probiotics were different, so we did not conduct publication bias assessment and subgroup analysis. In addition, the sensitivity analysis of the number of bowel movements per week was stable, but the results should be interpreted carefully. However, the advantages of our study are that (1) This is the first meta-analysis and systematic review of existing evidence to clarify the efficacy and safety of probiotics for constipation in PD patients, which will provide favorable evidence for evidence-based medicine. (2) The reviewers discussed the limitations of the included studies and proposed specific suggestions for future studies to provide reliable research results.

## Conclusion

Although the evidence in this systematic review only supports the notion that probiotics have a significant effect on increasing the number of bowel movements per week in patients with PD constipation, probiotics have potential value in the treatment of PD-related constipation based on the overall results of existing clinical observational studies, animal research reviews, and clinical experience.

## Author contributions

FZ and SY: study conception and design, data analysis, interpretation, and manuscript writing. All authors: final approval of manuscript.

## Acknowledgments

Thanks to LYL, DYC and ZL for their contribution to the data collection and assembly of this manuscript.

## Conflict of interest

The authors declare that the research was conducted in the absence of any commercial or financial relationships that could be construed as a potential conflict of interest.

## Publisher’s note

All claims expressed in this article are solely those of the authors and do not necessarily represent those of their affiliated organizations, or those of the publisher, the editors and the reviewers. Any product that may be evaluated in this article, or claim that may be made by its manufacturer, is not guaranteed or endorsed by the publisher.
