# Supplementary material for: Probiotics for constipation in Parkinson’s: A systematic review and meta-analysis of randomized controlled trials
Source: Front Cell Infect Microbiol. 2022 Nov 10;12:1038928. doi: 10.3389/fcimb.2022.1038928 (PMC9684193; doi:10.3389/fcimb.2022.1038928)

Excluded list for the 'Full-text assessed for eligibility'.

Citation	Reason for exclusion
Cassani E, Privitera G, Pezzoli G, et al. Use of probiotics for the treatment of constipation in Parkinson's disease patients. Minerva Gastroenterol Dietol. 2011;57(2):117-121.	No control group
Chong KK, Lim SY, Manap MAAA, Lim JL, Low SC, Mahadeva SK, et al. A randomized double-blind placebo-controlled trial of probiotics for constipation in Parkinson's disease. Movement Disorders (2018) 33:S194.	Incomplete data
Cassani E, Privitera G, Pezzoli G, Pusani C, Madio C, Iorio L, et al. Use of probiotics for the treatment of constipation in Parkinson's disease patients. Minerva Gastroenterologica e Dietologica (2011) 57(2):117-21.	Incomplete data
Cereda E, Pacchetti C, Bolliri C, Cassani E, Iorio L, Pusani C, et al. Double-blind, placebo-controlled trial of a fermented milk containing multiple probiotic strains and prebiotic fiber for constipation associated with Parkinson's disease. Movement Disorders (2016) 31:S99. doi: 10.1002/mds.26688.	Incomplete data
Machado D, Honeycutt L. Effects of probiotics on constipation, neurological symptoms, and quality of life associated with parkinson's disease. Neurology (2018) 90(15).	Incomplete data

Michela B, Pacchetti C, Bolliri C, Cassani E, Iorio L, Pusani C, et al. Double blind, placebo-controlled trial of a fermented milk containing multiple probiotics strains and prebiotic fiber for constipation associated with parkinson's disease. Journal of the Neurological Sciences (2015) 357:e260. doi: https://doi.org/10.1016/j.jns.2015.08.917.	Incomplete data

Database	Search strategy
PubMed	((probiotics[MeSH]) OR (Synbiotics[MeSH]) OR (Saccharomyces[MeSH]) OR (Escherichia[MeSH]) OR (Bifidobacterium[MeSH]) OR (Bacillus[MeSH]) OR (Lactobacillus[MeSH]) OR (Clostridium[MeSH]) OR (probiotics)) AND ((parkinson disease[MeSH]) OR (Parkinson's disease[Text Word]) OR (Parkinson's disease) OR (parkinson disease)) AND ((constipation[MeSH]) OR (difficult defecation[Text Word]) OR (constipation)) AND ((randomized controlled trial[Publication Type]) OR (clinical trial) OR (efficacy) OR (trial) OR (randomized controlled trial))
Embase	(Synbiotics:ti,ab,kw OR Saccharomyces:ti,ab,kw OR Escherichia:ti,ab,kw OR Bifidobacterium:ti,ab,kw OR Bacillus:ti,ab,kw OR Lactobacillus:ti,ab,kw OR Clostridium:ti,ab,kw OR probiotics:ti,ab,kw) AND (parkinson disease:ti,ab,kw OR Parkinson's disease:ti,ab,kw OR Parkinson's:ti,ab,kw) AND (constipation:ti,ab,kw OR difficult defecation:ti,ab,kw) AND (randomized controlled trial:ti,ab,kw OR clinical trial:ti,ab,kw OR efficacy:ti,ab,kw OR trial:ti,ab,kw)
The Cochrane Central Register of Controlled Trials	(Probiotics OR Synbiotics OR Saccharomyces OR Escherichia OR Bifidobacterium OR Bacillus OR Lactobacillus OR Clostridium) AND (parkinson disease OR Parkinson's disease) AND (constipation OR difficult defecation) AND (randomized controlled trial OR clinical trial OR efficacy OR trial)
Web of Science	(((ALL=(Probiotics OR Synbiotics OR Saccharomyces OR Escherichia OR Bifidobacterium OR Bacillus OR Lactobacillus OR Clostridium)) AND ALL=(parkinson disease OR Parkinson's disease)) AND ALL=(constipation OR difficult defecation)) AND ALL=(randomized controlled trial OR clinical trial OR efficacy OR trial)

Sensitivity Analysis of the number of bowel movements per week.

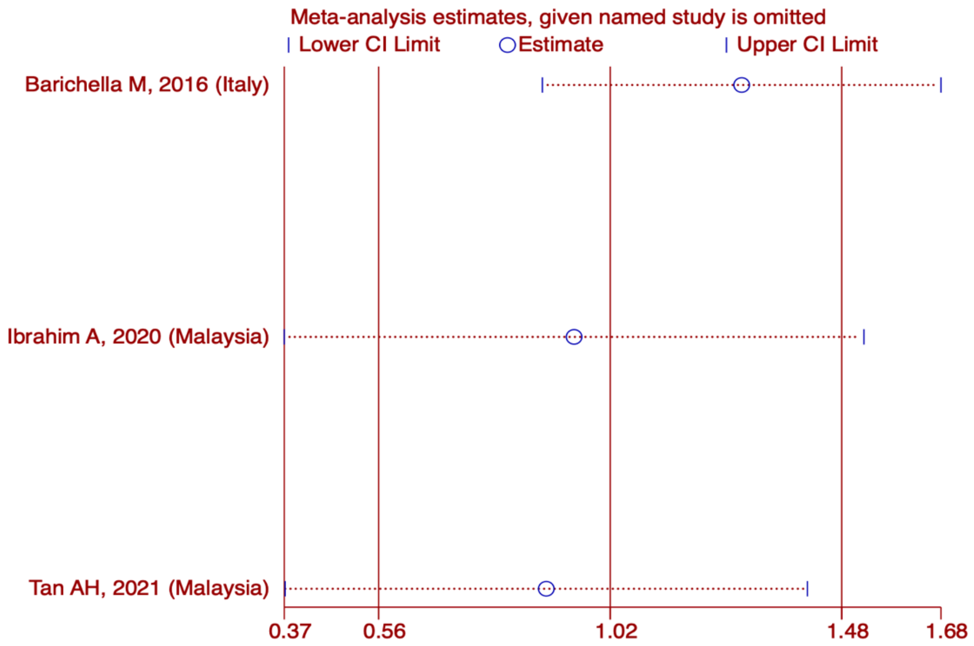

Supplement: Supplementary file 1 [file DataSheet_1.pdf]
